# A novel mouse model of postpartum depression using emotional stress as evaluated by nesting behavior

**DOI:** 10.1038/s41598-021-02004-9

**Published:** 2021-11-19

**Authors:** Tomoe Seki, Hirotaka Yamagata, Shusaku Uchida, Ayumi Kobayashi, Yoshifumi Watanabe, Shin Nakagawa

**Affiliations:** 1 Division of Neuropsychiatry, Department of Neuroscience, Yamaguchi University Graduate School of Medicine, 1-1-1 Minami-kogushi, Ube, Yamaguchi 755-8505 Japan; 2 Core Research for Evolutional Science and Technology (CREST), Japan Science and Technology Agency (JST), 4-1-8 Honcho, Kawaguchi, Saitama 332-0012 Japan; 3 Present Address: Seiwakai-Kitunan Hospital, 3381 Suzenji, Yamaguchi, 747-1221 Japan; 4 Present Address: SK Project, Medical Innovation Center, Kyoto University Graduate School of Medicine, 53 Shogoin-Kawahara-cho, Sakyo-ku, Kyoto, 606-8507 Japan; 5 Present Address: Southern TOHOKU Research Institute for Neuroscience, Southern TOHOKU General Hospital, 7-115 Yatsuyamada, Koriyama, Fukushima 963-8052 Japan

**Keywords:** Depression, Psychology and behaviour, Disease model, Experimental models of disease

## Abstract

Postpartum depression is an important mental health issue not only for the mother but also for the child’s development, other family members, and the society. An appropriate animal model is desired to elucidate the pathogenesis of postpartum depression. However, methods for stress loading during pregnancy have not been established. Behavioral experiments to investigate postpartum depression-like behaviors should be conducted without stress because behavioral tests affect rearing behaviors such as lactation. Therefore, we developed a new mouse model of postpartum depression using a psychological stress method. Mating partners were made to witness their partners experiencing social defeat stress and then listen to their cries. Emotional stress loading during pregnancy significantly increased postpartum depression-like behaviors. Postpartum depression also affected nurturing behaviors and caused disturbances in pup care. Furthermore, nesting behavior was impaired in the stressed group, suggesting that the observation of nesting behavior may be useful for assessing social dysfunction in postpartum depression. These results demonstrate the utility of this new mouse model of postpartum depression.

## Introduction

The prevalence of postpartum depression (PPD) is estimated to be approximately 10%, and PPD is a major problem in perinatal mental health^1^. PPD affects the mother, the infant, the partner, other family members, work, caregiving, and society^2^. The mother with PPD may engage in self-harm and suicide^2–4^, and there are also concerns regarding the effects on the infant, such as reduced attachment between mother and child and impaired long-term emotional, social, and cognitive development^2,5^.

Psychological stress during pregnancy has been reported as a risk factor for the onset of PPD in human studies^6^, and psychological care during pregnancy has been reported to prevent PPD^7–9^. To elucidate the pathophysiology of PPD in the brain, it is important to create animal models of PPD that mirror PPD in humans. However, the existing mouse models of depression are subjected to physical stress, including restraint stress^10^ or social defeat stress^11^, which may be too severe for pregnant mice. They are not appropriate as a model of PPD development in humans. Although there are several reports on the construction of rodent models of depression using non-physical stress^12–14^, it is unclear whether emotional stress during pregnancy induces PPD in mice.

For the analysis of anxiety and depression-like behavior in mice, the forced swim test, open field test, social interaction test, and sucrose preference test are generally applied^15,16^. However, these tests may expose the mice to novel and unpleasant environments, and there are concerns that the behavioral tests themselves may cause stress. In addition, the diagnosis of depression in the Diagnostic and Statistical Manual of Mental Disorders, Fifth Edition requires that in humans, significant clinical distress or impairment in social, occupational, or other important areas of functioning must have occurred due to depressive symptoms^17^. However, it may be challenging to assess murine functional impairment using these common behavioral tests. For example, the forced swim test, which is often used to screen for antidepressants, is not daily occurrence for mice, and immobility time is unlikely to be evaluated as an impairment in social activity. The social interaction test, commonly used for depressive behavior, may assess some aspects of social functioning. However, this test observes behavior in a novel environment and may not be sufficient to assess social functioning in daily life. Behavioral experiments to detect impairment in murine function equivalent to housework and childcare in humans that do not apply additional stress to evaluate a PPD model are desirable.

This study aimed to determine whether loading emotional stress, but not physical stress, to female mice, would increase their PPD-like behavior, including impairment of nesting and grooming of offspring, immobility on the forced swim test, and anxiety including open-field behavior.

## Results

Eight of 20 female mice in the control group and 7 of 20 female mice in the emotional stress group became pregnant and gave birth. Stillbirths or muricides were observed in 3 of 8 mothers in the control group and 3 of 7 mothers in the stress group. The average number of pups from the eight mothers in the control group was 5.0 ± 0.6 (mean ± standard error), and the average number of pups from the seven mothers in the stress group was 3.3 ± 0.6. There was no significant difference in the number of stillbirths and muricides (*p* = 0.86) or pups (*p* = 0.073) between the two groups (Table 1). Pups from four mothers in the control group (n = 18) and those from four mothers in the stress group (n = 11) were weighed, and no significant differences were found between the two groups (control group: 1.56 ± 0.06 g, stressed group: 1.68 ± 0.04 g, *p* = 0.18).

**Table 1:** The number of live or dead pups.

	Number of live pups	Number of dead pups
Control (n = 8)	5.0 ± 0.6	0.5 ± 0.3
Stress (n = 7)	3.3 ± 0.6	0.4 ± 0.3

Data are shown as the mean ± standard error. There was no significant difference between the two groups.

**Figure 1:**
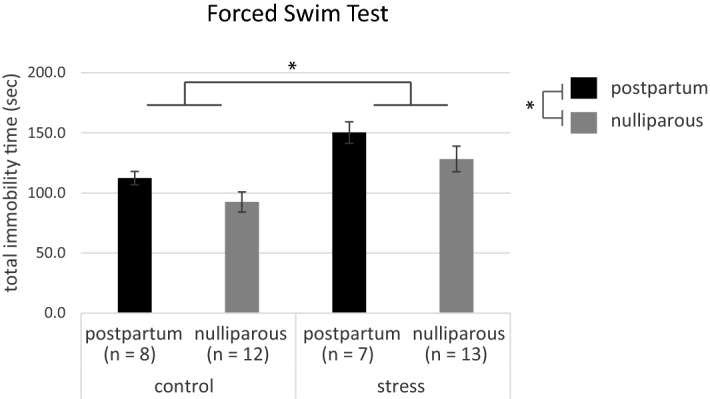
Immobility time on the forced swim test. Data are shown as the mean ± standard error. *p < 0.05 (two-way analysis of variance) for control versus stress and postpartum versus nulliparous.

The immobility time on the forced swim test was 112.4 ± 5.6 s for the postpartum mice and 92.5 ± 8.4 s for the nulliparous mice in the control group, and 150.3 ± 8.9 s for the postpartum mice and 128.2 ± 10.6 s for the nulliparous mice in the stress group (Fig. 1). Univariate two-way analysis of variance (ANOVA) revealed a main effect of stress and postpartum (stress: *p* = 5.7e−04, postpartum: *p* = 0.038) and no interaction.

**Figure 2:**
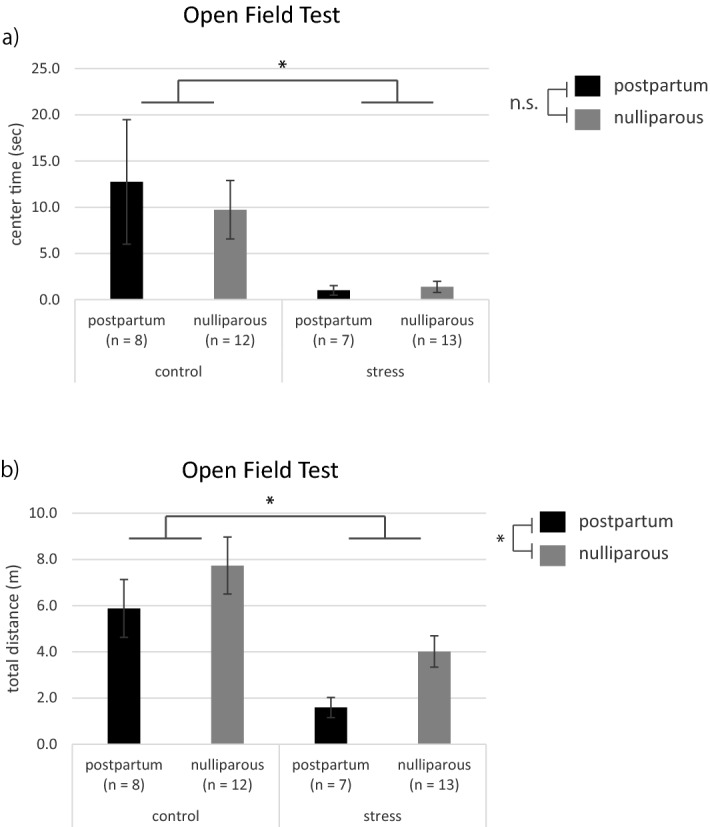
Comparison of the center time and total distance on the open field test. Data are shown as the mean ± standard error. (**a**) Center time, *p < 0.05 (two-way analysis of variance) for control versus stress. (**b**) Total distance, *p < 0.05 (two-way analysis of variance) for control versus stress and postpartum versus nulliparous. *n.s.* Not significant.

The center time on the open field test was 12.8 ± 6.7 s for the postpartum mice and 9.7 ± 3.2 s for the nulliparous mice in the control group, and 1.0 ± 0.5 s for the postpartum mice and 1.4 ± 0.6 s for the nulliparous mice in the stress group (Fig. 2a). Univariate two-way ANOVA indicated a main effect of stress (*p* = 0.0058), no effect of postpartum, and no interaction. The total distance was 5.9 ± 1.3 s for the postpartum mice and 7.7 ± 1.2 s for the nulliparous mice in the control group, and 1.6 ± 0.4 s for the postpartum mice and 4.0 ± 0.7 s for the nulliparous mice in the stress group (Fig. 2b). Univariate two-way ANOVA revealed a main effect of stress and postpartum (stress: *p* = 5.0e−04, postpartum: *p* = 0.048) and no interaction.

**Figure 3:**
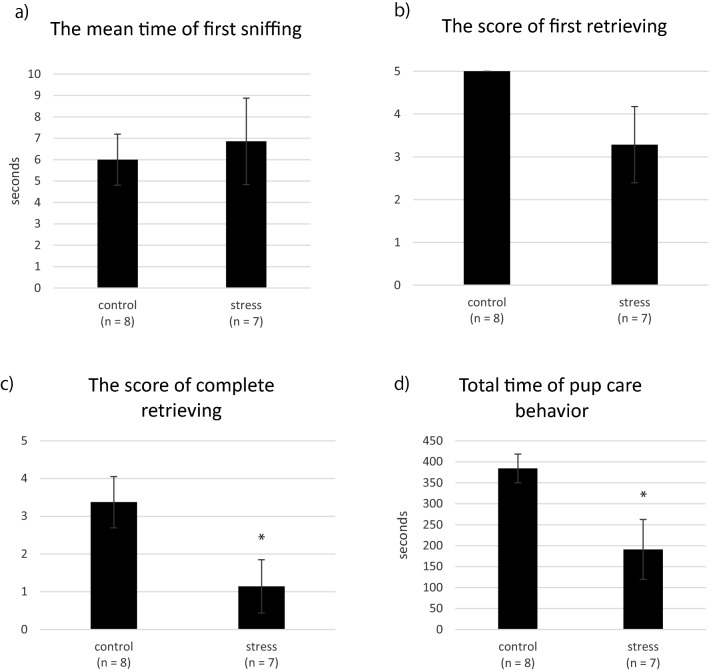
Comparison of nurturing behaviors. Data are shown as the mean ± standard error of the mean. The time of first sniffing (**a**), score of first retrieval (**b**), score of complete retrieval (**c**), and total time of nurturing behavior (d) in the control and stress groups of postpartum mice are shown. *p < 0.05 (t-test). *n.s.* Not significant.

Nurturing behavior was evaluated in eight control and seven mice in the stress group postpartum. The mean time of first sniffing was 6.0 s in the control group and 6.8 s in the stress group, with no significant difference (Fig. 3a). Two of the seven mice in the stress group failed to start retrieving any pups to the nest, and four mice did not retrieve any pups to the nest within 10 min. The mean score of first retrieval was 5.0 ± 0.0 in the control group and 3.3 ± 0.9 in the stress group, and the difference was not significant (Fig. 3b). The score of complete retrieval was 3.4 ± 0.7 in the control group and 1.1 ± 0.7 in the stress group; this score was significantly lower in the stress group than in the control group (*p* = 0.041) (Fig. 3c). The total time of pup care behavior was 384 ± 34 s in the control group and 191 ± 71 s in the stress group, and this score was also significantly lower in the stress group than in the control group (*p* = 0.024) (Fig. 3d).

**Figure 4:**
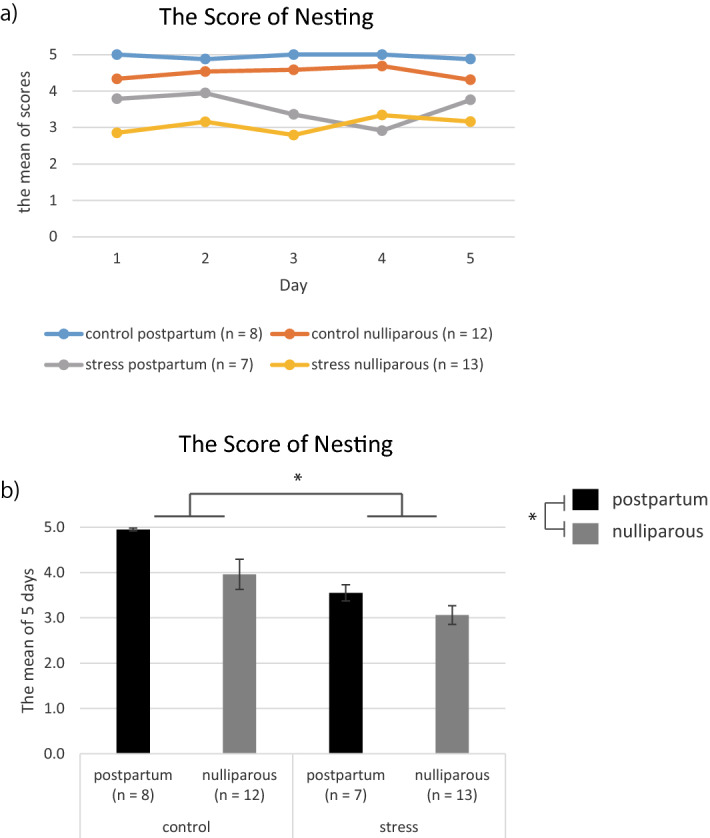
Comparison of nesting behaviors. Data are shown as the mean ± standard error. (**a**) Changes in nesting behavior scores in the four groups over time. (**b**) The mean scores for 5 days in the four groups. *p < 0.05 (two-way analysis of variance) for control versus stress and postpartum versus nulliparous.

The mean scores of the 5-day nesting evaluation were 4.9 ± 0.1 for the postpartum mice and 4.5 ± 0.2 for the nulliparous mice in the control group, and 3.6 ± 0.3 for the postpartum mice and 3.1 ± 0.2 for the nulliparous mice in the stress group (Fig. 4a,b). Univariate two-way ANOVA indicated a main effect of stress and postpartum (stress: *p* = 8.3e−05, postpartum: *p* = 0.0071) and no interaction.

## Discussion

In this study, it was found that perinatal emotional stress increased depressive behavior in female mice. We also found that the postpartum mice exhibited greater increases in depressive behavior than did the nulliparous mice. Furthermore, perinatal emotional stress reduced nurturing and nest-building behavior. These results indicate that our model is reflective of PPD in humans, in which psychological stress during the perinatal period leads to depression and impairments in parenting and social functioning.

Most of the existing models of PPD were based on the study of chronic stress, including physical stress^18–20^ and gestational stress^21–24^. In addition, stress loading is often performed before pregnancy^18,25^ because physical stress loading may be severe during pregnancy. A recent study showed that isolated mice exhibited increased depression-like behavior than group-housing mice^26^, supporting the notion that psychological stress can induce depression even without physical stress loading.

The first advantage of the present study is that PPD was observed with emotional stress but without physical stress. Physical stress-loading models could not completely rule out the possibility of behavioral abnormalities due to physical injury. Our stress-loading strategy can be applied when physical disability is a concern, such as using nude mice. Recently, it was reported that videos of social defeat stress caused overactivity in the hypothalamic–pituitary–adrenal axis and reduced reward sensitivity. However, no other depressive or anxiety symptoms, including those measured by the forced swim and social interaction tests, were observed^13^. In the present study, the postpartum mice were allowed to directly observe social defeat stress, resulting in depression-like behavior and social dysfunction. It may be necessary to determine whether PPD can be induced by video or sound alone to avoid excessive stress and cumbersome manipulation.

The second advantage is that both stresses during pregnancy and the effects of parturition were analyzed simultaneously. To the best of our knowledge, few studies have analyzed the interaction between parturition and stress during pregnancy. We have demonstrated that stress and parturition themselves increase depression-like behavior. The study’s observation of no interaction effect on depression-like behaviors between stress and parturition suggests that each is an independent factor increasing depressive-like behavior. This result may well reflect the clinical outcome of perinatal depression and may further emphasize the importance of perinatal mental health.

The third advantage is that we examined depression-like behaviors as well as nurturing behaviors and social functioning. Emotional stress loading reduces nurturing behaviors, which may affect the neural development of the offspring^27^. Further, it is well-established that depression-like behaviors in mice are often tested to evaluate depressive symptoms, but few studies have assessed functional impairment using versatile behavioral experiments. This study found that perinatal emotional stress impaired nest-building behavior in postpartum and nulliparous mice. Nest-building can be observed even in a single male mouse. It is believed that this behavior reflects environmental maintenance functions, such as cleaning and general housework in humans. It has been reported that acute stress and chronic social defeat stress impair nesting behavior^28–30^. The observation of nest-building behavior is expected to become a central behavioral assessment method for evaluating PPD-like behavior because it can be used to observe the nurturing behavior of mice without unnecessary stress loading.

However, there are several limitations to this study. The first is the use of BALB/c mice, which are known to be vulnerable to stress. Previous reports have demonstrated that differences in strain can lead to differences in PPD-like behavior^18^. Second, we did not examine the effects of therapeutic interventions. Third, we did not analyze the behavior of the offspring. Previous studies have shown that disturbances in nurturing behavior cause stress vulnerability in the offspring^27^. Finally, we did not analyze the pathogenesis of PPD, including hypothalamo-pituitary-adrenal axis. According to previous reports, the emotional stress of a single session in male C57BL/6J mice did not significantly alter corticosterone levels compared to controls^12^. Whether repeated emotional stress increases corticosterone levels is still unknown.

To address the above issues, we will conduct gene expression analysis in the brain of our new mouse model of PPD along with cytokine measurement, evaluation of the effects of antidepressant administration, and behavioral analysis of the offspring, which will help elucidate the pathogenesis of PPD and advance our understanding of interventions for perinatal health.

## Materials and methods

### Animals

Female and male BALB/c mice were acquired (Charles River Laboratories Japan Inc., Yokohama, Japan). The female mice were 8–9 weeks old, and their male partners were 10–11 weeks old at the time of experimentation. In addition, retired breeder CD1 male mice (Charles River Laboratories Japan Inc., Yokohama, Japan) were used as aggressors in the social defeat stress procedure applied to male mice. Only the BALB/c female mice were included in the analysis. All mice were maintained on a 12/12-h light/dark cycle with mouse chow and water provided ad libitum. All experimental protocols were approved by the Ethics Committee for Animal Experimentation of Yamaguchi University School of Medicine (approval number 20-113). All procedures were conducted in accordance with the ARRIVE guidelines and the Guidelines for Animal Care and Use at Yamaguchi University Graduate School of Medicine.

### Emotional stress procedure and evaluation

**Figure 5:**
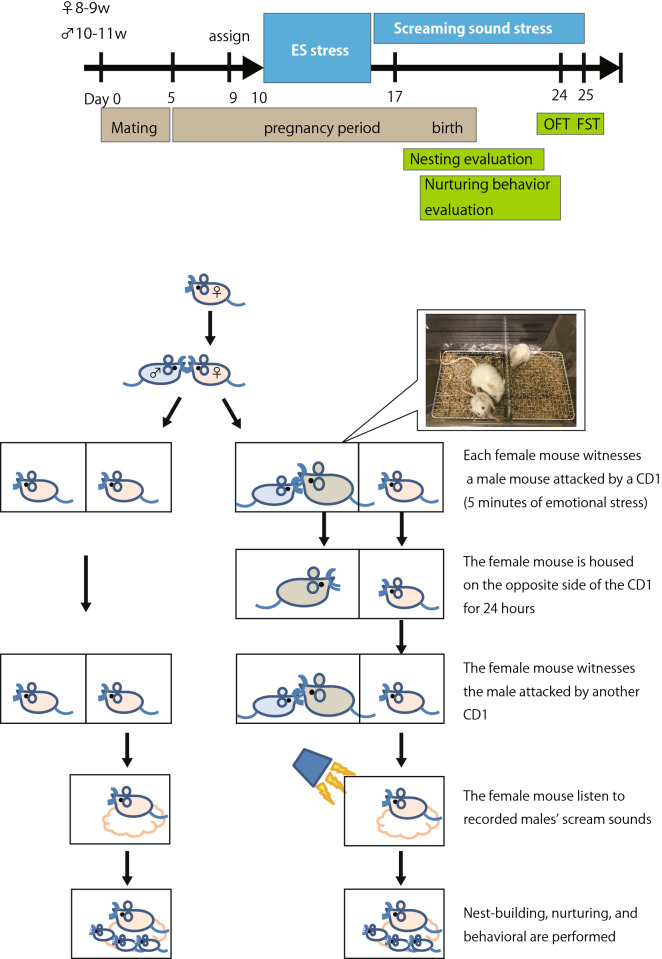
Procedure for the behavioral experiments. Female mice were housed together for mating (day 0). On day 5, the females were kept individually in a stress cage. Each female mouse witnessed a male mouse being attacked by a CD1 aggressor for 5 min on the other side of the cage. Following the fights, the female mouse was housed on the opposite side of the CD1 aggressor for 24 h. This session was repeated over 7 consecutive days. The female mouse was transferred to a normal cage and listened to recorded male mouse vocalizations during fighting for 10 h per day from day 17 to day 25. Nest building behavior was evaluated from day 19. The open field test (OFT) and forced swim test (FST) were performed on days 24 and 25, respectively.

The procedure for the behavioral experiments is shown in Fig. 5. The female mice were divided into 20 mice in the stress group and 20 in the control group. A total of 20 pairs, one male and one female, were housed together for mating. After co-housing for 4–5 days, females were kept individually in a stress cage (18.8 cm width × 29.6 cm depth × 12.8 cm height). The stress cage was divided into two compartments by a wire mesh in the center. Female mice were weighed daily from the end of mating until the day before the start of the stress experiment and were assigned to the control group or the stress group such that their weight gain would be equal. The males were kept in groups of 4–5 until the start of stress loading.

Emotional stress loading was performed using the modified chronic social defeat stress procedure as previously described^11,31,32^. Retired male breeder CD1 mice were used as aggressors and housed in the social defeat cage 48 h before the start of defeats on one side of the cage separated by a fine mesh divider. On the first day of stress, the BALB/c female mouse was placed on the other side of the cage from the CD1 aggressor. The BALB/c male mouse was placed on the same side of the cage as the CD1 aggressor for 5 min/day. During this time, the BALB/c male mouse was physically attacked by the unfamiliar CD1 aggressor, and the BALB/c female mouse witnessed the fights from the other side of the cage. Following the fights, the BALB/c male mouse was removed, and the female was housed on the opposite side of the divider, where it remained in sensory contact with the CD1 aggressor over a 24 h period. This session was repeated over 7 consecutive days, and the male BALB/c mouse was then exposed to a new CD1 aggressor in a different defeat cage. In preliminary experiments, male mice showed depression-like behavior after 7 days of this stress loading (Supplementary Fig. S1 online). The sound of the fight in the social defeat cage was recorded. In the control group, two BALB/c female mice were housed in the same social defeat cage separated by a fine mesh divider for the length of the stress period and rotated daily in a similar manner without being exposed to the CD1 aggressor.

After 7 days of stress loading, the females were transferred to normal cages and kept individually, and 8 g of Enviro-dri (Shepherd Specialty Papers, Inc., Milford, NJ, USA) nesting materials were introduced 12 h later. The recorded sound of the fight played for 6–12 h a day at random times, and the volume was set to a maximum of 75 dB to avoid hearing damage^33^. The recorded sound contained 8 min and 28 s of male mouse vocalizations from 12 min and 16 s of recorded material. The sound was played back repeatedly for 6–12 h, i.e., the actual duration of the sound was 4–8.2 h. Nest building evaluation commenced 2 days after the nesting material was introduced (19 days after the start of mating). Nest-building evaluation and confirmation of the presence or absence of birth were conducted between 8:00 and 10:00 a.m. If the birth was confirmed, nurturing behavior evaluation was also conducted during the abovementioned period. Behavioral experiments were conducted between 9:30 a.m. and 12:00 p.m. The open field test and forced swim test were performed on days 24 and 25 after the start of mating, respectively.

### Open field test

The open field test was performed by modifying the methods used in a previous study^34^. The test mice were placed in an open arena (30 cm length × 30 cm width × 16 cm height). The total length of the path the mouse traveled (locomotor activity) and the time it spent in the 10 × 10 cm center square (% center) was measured for 5 min using ANY-maze Video Tracking Software (Muromachi Kikai Co., Ltd. Tokyo, Japan).

### Forced swim test

The forced swim test was performed by modifying the methods used in a previous study^32^. Mice were placed in a water tank (20 cm height × 14 cm diameter filled with 23 ± 0.5 °C water to a depth of 13 cm) for 5 min, and the duration of floating (i.e., the time during which the mouse only performed the small movements necessary to keep its head above water) was measured.

### Nurturing behavior assessment

With reference to previous reports^35^, nurturing behaviors were assessed as follows: the assessment of nurturing behavior was conducted between 8:00 and 10:00 a.m. on the day of birth. The mother was removed from the cage, and three healthy-looking milk-banded pups were placed in the three corners away from the nest. For mothers with three or fewer births, all pups were placed in the corners; for mothers with four or more births, three of the pups were selected and placed in the corners. The mother was then promptly returned to the cage, and her behavior was observed for 10 min. Sniffing time (time to first approach and sniff the pups), first retrieval time (time to start bringing a pup back to the nest), complete retrieval time (time to bring one pup back to the nest), and pup care behavior (nesting retrieval: bringing pups back to the nest, grouping: grouping pups in the nest, crouching: getting into the feeding position, licking: licking pups) were assessed. For the first retrieval time, since some mothers did not complete retrieval within the setting time (5 min), we awarded 5 points for completion within the first 60 s, 4 points for completion within 120 s, 3 points for completion within 180 s, 2 points for completion within 240 s, 1 point for completion within 300 s, and 0 points for completion beyond that time or failure to complete.

### Nest building evaluation

**Figure 6:**
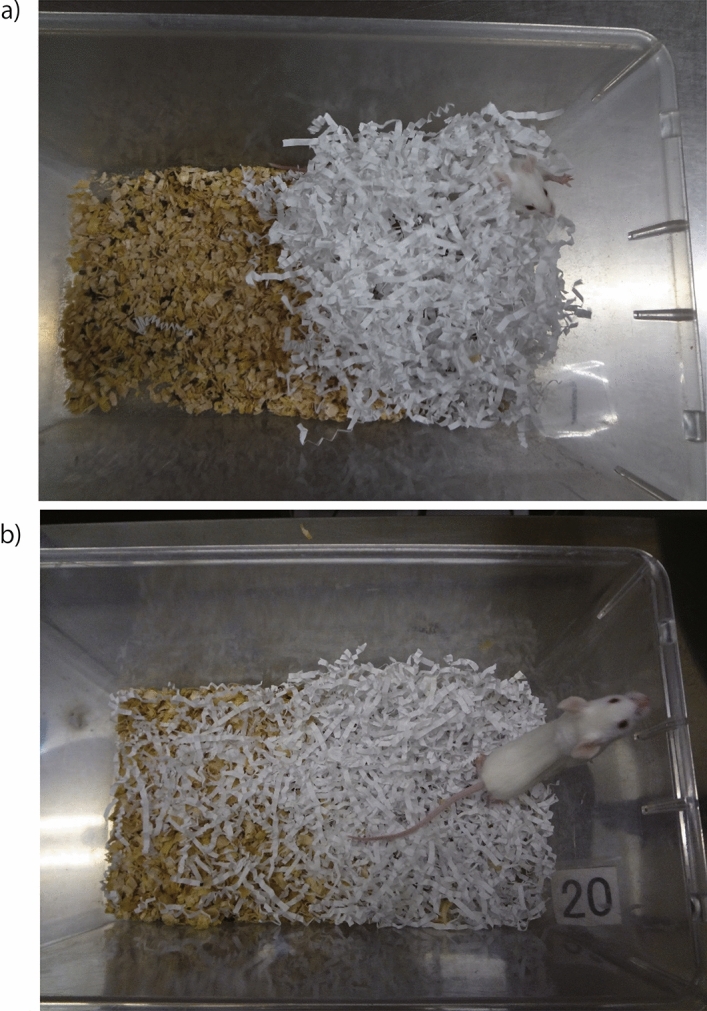
Examples of nesting assessment. (**a**) A perfectly domed nest (5 points). (**b**) A flat and cluttered nest (1.9 points).

We modified the method used in a previous report^36^ to evaluate nesting as follows: the evaluation of nest building was conducted between 8:00 and 10:00 a.m. on 5 consecutive days, ≥ 48 h after the introduction of nesting materials. The scores were calculated by recording the percentage of each score of all the nest materials (100%) as follows: 5 points for a fully domed nest (Fig. 6a), 4 points for an incompletely domed or semi-spherical nest cavity, 3 points for a cup or bowl nest, 2 points for a flat nest, 1 point for movement of nest materials but not collected as a nest, and 0 point for no movement of the nesting materials^36^. For example, if 90% of the nesting material in the cage was collected in a flat nest and 10% was not, the score was calculated as 2 points × 0.9 + 1 point × 0.1, for a total of 1.9 points (Fig. 6b).

### Statistical analysis

SPSS 16.0 (SPSS Inc., Chicago, Illinois, USA) was used for data analysis. Univariate two-way ANOVA was used for the open field test, forced swim test, and nesting evaluation in four groups: the control groups with and without pregnancy and birth and the stress groups with and without pregnancy and birth. For the birth rate, pups’ weight, and nurturing behavior, mice in the stress and control groups were compared using an independent sample t-test. *p* < 0.05 was considered to indicate statistical significance. Data are presented as the mean ± standard error of the mean. The statistical summary analysis of behavioral data is shown in Supplementary Table S1 online.

## Supplementary Information

Supplementary Figure S1.

Supplementary Table S1.

## Data Availability

All data in this published article (and its Supplementary Information files) are available upon request.
