# Supplementary material for: A novel mouse model of postpartum depression using emotional stress as evaluated by nesting behavior
Source: Sci Rep. 2021 Nov 19;11:22615. doi: 10.1038/s41598-021-02004-9 (PMC8604943; doi:10.1038/s41598-021-02004-9)
Supplement: Supplementary file 1 — Supplementary Figure S1. [file 41598_2021_2004_MOESM1_ESM.pdf]

Supplementary Figure S1

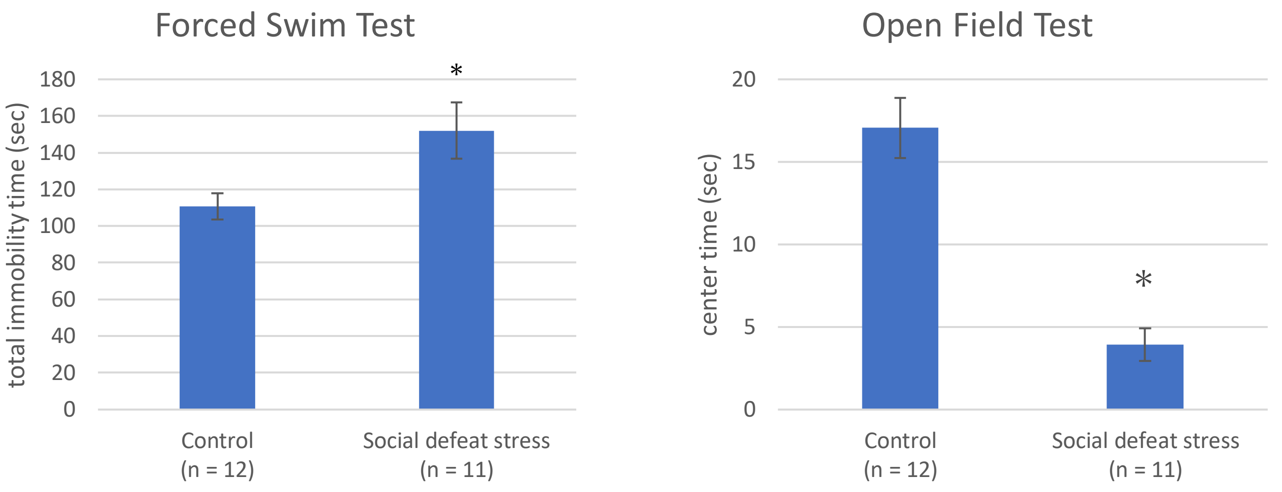

Figure S1. Depression-like behavior in male mice after social defeat stress
Data are shown as the mean ± standard error. *$p<0.05$ (independent sample t-test) for control versus stress.
