# Supplementary material for: A novel mouse model of postpartum depression using emotional stress as evaluated by nesting behavior
Source: Sci Rep. 2021 Nov 19;11:22615. doi: 10.1038/s41598-021-02004-9 (PMC8604943; doi:10.1038/s41598-021-02004-9)
Supplement: Supplementary file 2 — Supplementary Table S1. [file 41598_2021_2004_MOESM2_ESM.docx]

| Behavioral paradigm | Measurement | Statistical test | Comparison | Statistics | p-value | Fig. |
| --- | --- | --- | --- | --- | --- | --- |
| Forced swim test | Immobility time | Two-way ANOVA | Factor1: stress | F(1,36) = 14.27 | p = 5.7e-04 | 1 |
|  |  |  | Factor2: postpartum | F(1,36) = 4.63 | p = 0.038 |  |
|  |  |  | Interaction (F1 x F2) | F(1,36) = 0.01 | p = 0.91 |  |
| Open field test | Center time | Two-way ANOVA | Factor1: stress | F(1,36) = 8.62 | p = 0.0058 | 2a |
|  |  |  | Factor2: postpartum | F(1,36) = 0.15 | p = 0.70 |  |
|  |  |  | Interaction (F1 x F2) | F(1,36) = 0.25 | p = 0.62 |  |
|  | Total distance | Two-way ANOVA | Factor1: stress | F(1,36) = 14.64 | p = 5.0e-04 | 2b |
|  |  |  | Factor2: postpartum | F(1,36) = 4.18 | p = 0.048 |  |
|  |  |  | Interaction (F1 x F2) | F(1,36) = 0.07 | p = 0.79 |  |
| Nurturing behavior | Time of first sniffing | Unpaired t-test |  | t = -0.38 | p = 0.073 | 3a |
|  | Score of first retrieval | Unpaired t-test |  | t = 2.07 | p = 0.059 | 3b |
|  | Score of complete retrieval | Unpaired t-test |  | t = 2.27 | p = 0.041 | 3c |
|  | Total time of childcare behavior | Unpaired t-test |  | t = 2.55 | p = 0.024 | 3d |
| Nesting | Score of nesting | Two-way ANOVA | Factor1: stress | F(1,36) = 19.69 | p = 8.3e-05 | 4b |
|  |  |  | Factor2: postpartum | F(1,36) = 8.15 | p = 0.0071 |  |
|  |  |  | Interaction (F1 x F2) | F(1,36) = 0.92 | p = 0.34 |  |

**Table S1.** **Statistical summary analysis of behavioral data**
